# The influence of chemical protection on the content of heavy metals in wheat (*Triticum aestivum* L.) growing on the soil enriched with granular sludge

**DOI:** 10.1007/s10661-017-6143-8

**Published:** 2017-07-31

**Authors:** E. Wołejko, B. Łozowicka, P. Kaczyński, R. Konecki, M. Grobela

**Affiliations:** 1 Department of Chemistry, Biology and Biotechnology, Białystok University of Technology, Wiejska 45 E, 15-351 Białystok, Poland; 2 Laboratory of Pesticide Residues, Institute of Plant Protection – National Research Institute, Chełmońskiego 22, 15-195 Białystok, Poland; 3 Institute of Plant Protection – National Research Institute, Węgorka 20, 60-318 Poznań, Poland

**Keywords:** Heavy metal, Granular sludge, Herbicide, Fungicide, Grain, Plant

## Abstract

The presence of heavy metals in *Triticum aestivum* L. growing on the soil enriched with granular sludge after chemical protection was observed. The five variants of treatments using herbicide (Chwastox Turbo 340SL) and four fungicides (Topsin M 500SC, Amistar 250SC, Artea 330EC, and Falcon 460EC) were performed. On control and experimental plots, the concentration of Ni, Pb, Cr, and Cu in wheat leaves were in the range 0.32–0.99, 0.92–1.57, 0.89–6.31, and 7.08–12.59 mg/kg and in grains 0.03 to 0.11, 0.14–0.25, 0.11–0.76, and 1.06–1.46 mg/kg, respectively. The concentration of Pb in grain protected by MCPA and 2,4-D with thiophanate-methyl and azoxystrobin was higher than the maximum levels of 0.20 mg/kg D.M. The bioconcentration factor (BCF) differed and depended on chemical protection. The highest value of BCF was achieved for Cd. The statistical analysis showed a significant correlation between concentration of metals and quality parameters of wheat. One observed significant negative correlations between Ni/Zeleny sedimentation value (*r* = −0.51) and between Pb/starch content (*r* = −0.57). Positive correlations were observed between Cd/yield, the number of grains/ergosterol concentration (respectively, *r* = 0.41, *r* = 0.55, *r* = 0.56), and Zn/thousand grain weight (*r* = 0.50) at a *p* ≤ 0.05.

## Introduction

Wheat (*Triticum aestivum* L.) is one of the most popular cereals cultivated in the world, holding a large yield potential. Versatile use in food, feed, and pharmaceutical field forces the production of high quality and quantity yield (Singh et al. 2010).

Wheat belongs to plants susceptible to attacks of fungal pathogens, pests, and weeds, which lead to significant yield losses (Łozowicka et al. 2012). The severity of their occurrence is related to climatic conditions, monoculture crop with the simplification of cultivation methods, or ineffective protection (Bjorling-Poulsen et al. 2008).

In wheat agrophage control, one commonly uses agronomic, biological, mechanical, and chemical methods; the last provides extremely effective protection. Used plant protection products contribute not only to inhibiting the growth of pathogens, pests, and weeds but also to stimulating the growth, the presence of assimilation pigments, and yield of plants (Balba 2007; Łozowicka et al. 2016).

Modern fungicides used in wheat protection on the basis of strobilurins, triazoles, and amines inhibit the growth of the fungus through the stop electron transfer between cytochrome b and cytochrome c, which reduces oxidation of nicotinamide adenine dinucleotide (NADH) and adenosine triphosphate (ATP) synthesis (Balba 2007; Rosales-Conrado 2009) as well as inhibits sterol biosynthesis (Jabłońska-Trypuć et al. 2017).

Among the large group of herbicides utilized in wheat production, MCPA and dicamba (Pernak et al. 2011) are still being popular, in combination or either alone. These compounds provide broadleaf weed control. From another point of view, they may negatively influence crop and humans (Pernak et al. 2011). These herbicides may be characterized by resistance to biodegradation processes and they may influence the amount of specific microbial species, thus the growth and quality of crops (Ławniczak et al. 2016).

In the agronomic methods, one of the innovative solutions to improve quality parameters of soil sediments is using an alternative source of organic matter and nutrients (Birkhofer et al. 2008). The sewage sludge may be an excellent product which makes it possible to recycle valuable components such as organic matter, nitrogen, phosphorus, and other nutrients for plants. Fertilization with stabilized settlements contributes to increasing the amount of humus in soil as well as to improving its quality. It may also facilitate increasing the yield of various crops such as cereals, vegetables, shrubs, and trees (Wuana and Okieimen 2011).

Searching for new solutions reducing the deficit of soil organic matter results from insufficient production of manure. Therefore, an interesting option is to use sewage sludge on crops where gradual releasing nutrients ensures plant growth at a satisfactory level (Zhao et al. 2001). On the other hand, in addition to the positive aspects of the favorable biological compounds, deposits can contain harmful and toxic compounds, e.g., heavy metals (Wołejko et al. 2014).

The application of sludge on production soil due to the stability of heavy metals in the environment can contribute to their inclusion into the biological cycle (Bose and Bhattacharyya 2008). Consequently, the plants absorbing soil nutrients can also assimilate heavy metals, which may lead to contamination of crops at concentrations exceeding permitted levels, which disqualifies them in terms of quality (Safari et al. 2015).

The heavy metals such as cadmium (Cd), lead (Pb), chromium (Cr), copper (Cu), and zinc (Zn) are an important group of trace elements, which in concentrations above the norm can negatively influence the development of people, animals (Cd and Pb), and plants (Zn and Cr) (Castaldi et al. 2009; Safari et al. 2015). Bioaccumulation of microelements in plants causes their inclusion in the food chain: soil-plant-animal-human. Metals occurring in the form of free ions are most easily accumulated from soil by plants. In turn, metals existing in complexes can be released from soil by active substances secreted by plant roots and microorganisms in soil, then easily taken up by plants, thus affecting the growth and quality of yield (Zaccone et al. 2010).

In the literature, there are many reports describing influence of heavy metals on soil degradation in industrial areas (Birkhofer et al. 2008; Castaldi et al. 2009; Singh et al. 2010). According to the best of our knowledge, there is a lack of research studies of agricultural soils and crops where the pesticide was applied and the evaluation of migration of heavy metals in the system soil-plant-grain was conducted.

We hypothesize that fungicides and herbicides may stop or activate migration of the heavy metals into the soil-plant-cereals system as well as they may also influence quality parameters of grain.

Therefore, the novelty of this study was to evaluate the effect of different chemical wheat protective treatments on migration of heavy metals present in the soil enriched with sewage sludge to plants and grains. Additionally, the aims were to evaluate the bioaccumulation factor and to search for the correlation between quantitative and qualitative parameters of wheat and metal content in the grain.

## Materials and methods

### The field experiment and weather conditions

The field experiment was conducted in 2014 in Podlaskie (53° 11′ 45.2″ N 23° 00′ 40.4″ E) using the method of randomized blocks with four replications. Spring wheat was sown on 31.03.2014. Fertilization had been performed before sowing (28.03.2014), using organic fertilizer (N 87.5 kg/ha, P 133 kg/ha, organic substance 1400 kg/ha). In addition, on the plots, one used sewage sludge from the local sewage treatment plant. According to Zadoks’ scale (Zadoks et al. 1974) in the shooting phase (BBCH 30), ammonium nitrate (N 34 kg/ha) was used.

Meteorological data were collected from the automatic weather station located near the experimental plots (53° 11′ 50.2″ N 23° 00′ 41.4″ E). In the conducted experiment, weather conditions were characterized by low rainfall, the sum of which amounted to 25 mm in April, 94 mm in May, 72 mm in June, and 74 mm in July. The observed amount of rainfall was lower on average by 38% with respect to rainfall in the same period (April–July) in previous years (2002–2013). Average daily temperatures in April, May, June, and July were respectively 8, 14, 13, and 20 °C.

### The variants of chemical protection

The following kinds of plots were set: the control plots without chemical protection or manual weed control and the plots where one applied chemical protection using a knapsack compressed air sprayer AP-1/p in five variants (Table 1). The treatments were performed using one herbicide and four fungicides (Table 1). The herbicide (Chwastox Turbo 340SL) composed of two active substances carboxylic acid group (MCPA) and benzoic acid derivatives (dicamba) was treated at tillering phase (scale BBCH 19-23). Fungicidal treatments were repeated twice at an interval of 2 weeks. In the wheat heading stage (BBCH 44-58), protective treatment was made using Topsin M 500SC compounds from the group of benzimidazoles (thiophanate-methyl) and Artea 330EC containing the active substances of triazole (propiconazole and cyproconazole). Fungicide application was performed using Amistar 250SC with active substance from the group of strobilurins (azoxystrobin) and Falcon 460EC with plant protection product consisting of spiroxamine, tebuconazole, and triadimenol in the stages of BBCH 71–77 (Table 1).

**Table 1:** The chemical protection variants of spring wheat

Treatment no.	Variants of the chemical protection	Active substance (a.s.)	Content a.s. (g/l)	Dose (l/ha)	Phase of growing plant BBCH^a^
1	Control	–	–	–	–
2	H	MCPA Dicamba	300 40	2.0	21–23
3	F1	Thiophanate-methyl	500	1.4	56–58
		Azoxystrobin	250	0.8	71–73
4	H	MCPA Dicamba	300 40	2.0	21–23
	F1	Thiophanate-methyl	500	1.4	56–58
		Azoxystrobin	250	0.8	71–73
5	F2	Propiconazole Cyproconazole	250 80	0.5	56–58
		Spiroxamine Tebuconazole Triadimenol	250 167 43	0.6	71–73
6	H	MCPA Dicamba	300 40	2.0	21–23
	F2	Propiconazole Cyproconazole	250 80	0.5	56–58
		Spiroxamine Tebuconazole Triadimenol	250 167 43	0.6	71–73

*H* herbicide, *F1* first set of fungicides, *F2* second set of fungicides

^a^BBCH according to (Zadoks et al. 1974)

### Parameters of wheat grain quality

The wheat grain samples from each plot were collected for the determination of protein content (%), wet gluten content (%), starch content (%), and ergosterol content (kg/ha). All samples were measured with NIR/NIT technology fitted with a sample transport module and standard sample cups with a grain analyzer (InfratecTM 1241, Foss). Moreover, one also determined thousand grain weight (TGW) (g), test weight (TW) (kg/hL), and Zeleny sedimentation volume (ZSV) (ml) using the AACC method (American Association of Cereal Chemists International, 2000).

### Chemical analyses of soil and sewage sludge

Laboratory studies included the determination of the total nitrogen concentrations in soil using the Kjeldahl method after mineralization of samples in concentrated sulfuric acid (Bremner and Mulvaney 1982), organic carbon was measured by the Tiurin method, and the content of total sulfur was determined by the nephelometric method of Butters and Chenery (1959). The values of pH in soil were measured by 1 M KCl.

### Analyses of heavy metals

Metal contents of samples were analyzed in an accredited laboratory, using certified reference materials INCT-MPH2. The samples of the plants (0.5 g) were digested in a closed microwave system (CEM MARS 5) in 10 ml of 65% HNO_3_ (Merck suprapur®). The samples were heated during digestion in two cycles by extending the time, increasing the temperature, and using the microwave oven at a fixed power level (1600 W, 100% for 5 min, the temperature increased to 135 °C, then for 5 min, it maintained at 135 °C; 1600 W, 100% for 5 min, the temperature increased to 165 °C, then for 20 min, it remained at 165 °C). After cooling, the samples were filtrated into 50-ml volumetric flasks. The total content of metals was analyzed by means of the atomic absorption spectrometry (AAS) by using Varian AA240Z – graphite furnace atomic spectroscopy (GFAAS). The accuracy of the completed mineralization and GFAAS determination was confirmed in the analysis of the certified material INCT-MPH2 (mixture of Polish herbs)—recovery percentage of the reference material was equal to 97.7%. All samples were analyzed in triplicate.

The samples of soils (0–25 cm) and soils with sewage sludge were collected and the concentrations of Cd, Cr, Cu, Ni, Pb, and Zn were determined by the AAS by using Thermo Scientific iCE3300. All the samples (1.0 g) were mineralized by using microwave digestion system and remains were dissolved in aqua regia at 80 °C in triplicate, according to the Polish norm PN-ISO 11047:2001. ERM-CD281 reference material was used to evaluate the correctness of the results.

In all analyses, one used water with conductivity 0.05 μS/cm from a Polwater CDRX-200 deionizer. All laboratory vessels used in the experiment were immersed in 10% HNO_3_ for 24 h and then rinsed with deionized water.

### Parameters of sewage sludge applied in the field experiment

The sludge was dried at 130 °C and formed in granules. It did not contain *Salmonella* or invasive nematode ova and was applied on the experimental plots at a dose of 3 kg/ha D.M. The concentration of heavy metals of sewage sludge is presented in Table 2. The sewage sludge used in the experiment had pH −7.5 and contained the following: dry matter 89%, organic matter 56.2 g/kg D.M., total N 4.6% D.M., ammonium N 0.32% D.M., total P 3.2% D.M., Mg 0.72% D.M., and Ca 4.16% D.M.

**Table 2:** The experiment and normative values of concentration of heavy metals of sewage sludge used in agriculture purposes

Heavy metal	Experimental concentration	Normative concentration
	(mg/kg D.M.)	
Pb	21.9	750
Cd	1.2	20
Cu	208	1000
Cr	64.3	500
Ni	30.01	300
Zn	1100	2500

*D*.*M*. dry matter

### Bioconcentration factor

The biological concentration factor (BCF) as given by Arnot and Gobas (2006) and Alam et al. (2003) for analyzed heavy metals was determined as quotients of average concentration of a given element in plants with relation to its average concentration in soil and was defined as

$$ BCF={C}_{\mathrm{plant}}/{C}_{\mathrm{soil}} $$

where

$$ {C}_{\mathrm{plant}}\hbox{---} total\  concentration\  of heavy\  metals\  in\  the\  above ground\  parts\  of\  plants\  in\ \mathrm{mg}/\mathrm{kg}\ \mathrm{D}.\mathrm{M}. $$

$$ {C}_{\mathrm{soil}}\hbox{---} \mathrm{total}\ \mathrm{concentration}\ \mathrm{of}\ \mathrm{heavy}\ \mathrm{metals}\ \mathrm{in}\ \mathrm{soil}\  in\ \mathrm{mg}/\mathrm{kg}\ \mathrm{D}.\mathrm{M}. $$

### Statistical analysis

The statistical analysis was performed by Statistica 13. The correlation between heavy metal concentration in the above ground parts of plants and in soil fertilized with sewage sludge was calculated using Spearman’s correlation factor *r* for *p* ≤ 0.05. Moreover, the results obtained for grain and plants were calculated statistically by means of variance analysis and the relevant differences were evaluated by Tukey’s test, on the relevance level of *α* = 0.05.

## Results and discussion

### The influence of sewage sludge on parameters of soil

In this study, sewage sludge was applied to cereal crops as an alternative source of nutrients necessary for proper development of plants. The applied sewage sludge influenced the parameters of the soil where wheat was sown and different variants of chemical protection were applied. Table 3 shows physical and chemical parameters of the soil prior to the application and enrichment of sludge treatment.

**Table 3:** Selected chemical properties of soil before and after application of sludge

	pH	N	C	S	Cu	Zn	Pb	Cd	Cr	Ni
		(% D.M.)			(mg/kg D.M.)					
Control soil (without sewage sludge)	6.6–7.0	0.15	1.45	0.026	6.7	24.0	8.59	0.13	8.2	nd
Soil enriched in sewage sludge	7.2–7.4	1.50	3.75	25.21	32	86.14	18.94	0.81	42.3	5.89
Normative maximum limit of content heavy metals in soil	–	–	–	–	50	120	60	2	75	35

*nd* not detected, *D*.*M*. dry matter

One analyzed heavy metal concentration in the soils before the experiment determining the contents of Zn, Cd, Ni, Cu, Pb, and Cr under the geochemical background. According to Lis and Pasieczna 1995, the natural content of heavy metals in soil in comparison with the geochemical background was Cr 5–10 mg/kg (8.2), Cu 20–40 mg/kg (6.7), Ni <5 mg/kg (nd), Cd <0.5 mg/kg (0.13), Pb 12.5–100 mg/kg (8.59), and Zn 25–400 mg/kg (24.0) (the average values of the individual heavy metal accumulation in the experimental soil before application sewage sludge given in parentheses).

According to the actual appropriate The Directive of Environmental Minister on municipal sewage sludge (J. of Laws, 2015, item. 257), the tested sludge, soil enriched with granular sludge and soil samples taken from the top layer of plots (0–25 cm) met these official criteria (Table 3).

The dynamics of changes in metal forms is most intense at the top layer of soil. It depends on the diverse population of microorganisms, organic matter content, the sorption capacity, biological interactions of microorganisms of the rhizosphere as well as the plant itself, and protective treatments performed (Walker et al. 2004). Metal accumulation in soil and their availability to plants are also determined by the pH of the soil. In our study, the pH ranged from 6.6 to 7.4, so the soil and heavy metals contained in it should not be a major threat to plants and the environment and therefore should not go into the food chain (Wołejko et al. 2014; Yoneyama et al. 2010). Moreover, Wang et al. (2006) claim that metal solubility is conditioned by the exchange sorption processes and it is low for neutral and alkaline reactions. In turn, as reported by Tarek and Shehata (2015), during growth of plants and root development, roots emit into the soil organic acids which may make heavy metal solubility more intensive and influence absorption through the root system. It is confirmed by own research, as in the grain collected from the control plots where the pH was neutral, one observed slightly exceeded lead content in comparison with the allowed given in the Commission Regulation (EU) No. 1881/2006. Whereas, according to Merrington and Smernik (2004), increasing soil pH by adding sewage sludge may also result in the releasing of some metals such as Cr from the soil and causing oxidation of Cr^3+^ to a more mobile and toxic Cr^6+^, which is taken up by plants and can go into the food chain (Sivakumar and Subbhuraam 2005).

### Influence of chemical treatments on migration of metals in plant and wheat grains

**Fig. 1:**
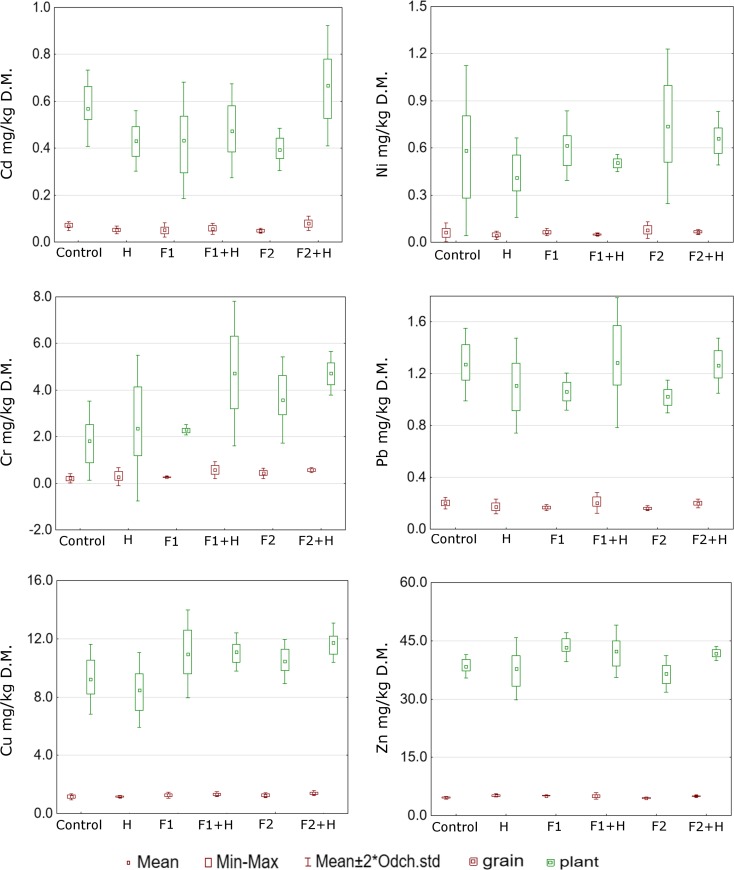
Metal concentration (mg/kg D.M.) in plant and wheat grains depending on the applied plant protection treatment

The results shown in Fig. 1 indicate that the application of the six variants of protection treatments influenced the migration of different heavy metals from the soil to plants. Defending themselves against poisoning by heavy metals, plants evolved defense mechanisms allowing them to grow and develop in contaminated environment. One of the most important components of the stress response induced by heavy metals is the synthesis of phytochelatins (Cobbett 2000). Based on these results, it was found that the average cadmium content in plants differed significantly depending on the procedure performed. The highest average cadmium content was recorded in plants collected from the plots where one used H + F2 in wheat leaves approximately 0.67 and 0.08 mg/kg D.M. in wheat grains, while the lowest was observed at the applied fungicide treatment F2 in leaves 0.39 and 0.047 mg/kg D.M. in wheat grains (Fig. 1, Table 4). Cadmium content in wheat grain was within the exposure limit values according to which the maximum level for Cd is 0.10 mg/kg D.M. (EU 1881/2006). Yoneyama et al. (2010) reported that some metals including cadmium can be absorbed by the clay minerals in the soil, thus lowering the concentration of metals in dissolved form and reducing the absorption of this element by plants (Merrington and Smernik 2004). Furthermore, as suggested by Zaccone et al. (2010), cadmium in the grain is distributed evenly and after elimination of bran, it remains in the flour in the same proportion as it appeared in the grain, thus threatening the health of animals and humans. It may induce small skeletal damage, kidney dysfunction symptoms, and reproductive deficiencies.

**Table 4:** Analysis of variance between concentration of each metal in plant/grain and chemical treatment (statistically significant for the *α* = 0.05)

	Cd	Cr	Cu	Ni	Pb	Zn
Grain						
Control	0.068	0.219	1.149	0.063	0.201	4.605
H	0.052	0.284	1.154	0.044	0.175	5.167
F1	0.052	0.276	1.259	0.066	0.168	5.093
F1 + H	0.057	0.565	1.331	0.051	0.203	5.076
F2	0.047	0.429	1.253	0.079	0.162	4.559
F2 + H	0.080	0.565	1.405	0.065	0.199	5.003
NIR	0.025	0.321	0.144	ns	ns	0.501
Plant						
Control	0.569	1.829	9.211	0.584	1.272	38.405
H	0.431	2.366	8.485	0.411	1.109	37.811
F1	0.434	2.300	10.948	0.616	1.061	43.328
F1 + H	0.474	4.715	11.098	0.504	1.285	42.333
F2	0.394	3.574	10.449	0.737	1.024	36.474
F2 + H	0.667	4.715	11.714	0.661	1.263	41.725
NIR	0.207	2.677	2.281	ns	ns	ns

*ns* non-significant

As observed by Garcia-Delgado et al. (2007), chromium is taken up by plants by passive transport because the plants do not accumulate chromium, even if it is present in large quantities, but in the environment, the concentrations of chromium is steadily increasing which is a matter of concern (Bielicka et al. 2005). In our study, the average chromium content ranged from 0.89 to 6.31 mg/kg D.M. in wheat leaves and from 0.11 to 0.76 mg/kg D.M. for wheat grains. Applied plant protection treatments significantly affected the absorption of Cr by wheat (Fig. 1, Table 4); the largest quantities of Cr were taken by plants growing on plots F1 + H and F2 + H, while the smallest amount was determined in the samples from the control plots.

Copper belongs to metals necessary for the proper conduct of some processes like the formation of proteins, photosynthesis, respiration, or the transformation of nitrogen compounds and permeability of cell membranes (Miotto et al. 2014). Based on these results, one observed that the highest statistically significant copper content occurred on the plots where treatments F1 and F2 + H were applied, while the smallest Cu was observed in plants collected from the plots where one applied only herbicide treatment (H) and this amount was lower by 8% compared with control plots. The average copper content ranged from 7.08 to 12.59 mg/kg D.M. in wheat leaves and from 1.06 to 1.46 mg/kg D.M. for wheat grains (Fig. 1, Table 4). Yruela (2005) points out that in soils, copper occurs in various forms, usually forming combinations of little mobility in the form of precipitates of carbonate and sulfate. Despite the low mobility, copper is easily absorbed by plants, thus resulting in the inhibition of enzyme activity or protein function, as well as a deficit of other essential ions through the disturbance of the cell transport (Yruela 2005; Wuana and Okieimen 2011).

In our study, one observed different contents of nickel in plants, depending on the plant protection treatment. The highest amount of nickel was taken by the plants from the plots with F2 fungicide treatment and from the plots where one applied MCPA and 2,4-D (H). The content of Ni in plants and grain was low and amounted to respectively 0.41 and 0.04 mg/kg D.M. (Fig. 1, Table 4). The average nickel concentration was in the range from 0.32 to 0.99 mg/kg D.M. in wheat leaves and from 0.04 to 0.11 mg/kg D.M. for wheat grains. Nickel, like copper and manganese, belongs to important micronutrients influencing plants’ growth and development, but at high concentrations, it is toxic and can affect the permeability of the membrane, inhibit sprouting, and limit the growth and development of plants, thus affecting yield reduction (Boominathan and Doran 2002). At the low pH, the bond strength of nickel by soil organic matter is small, and at the neutral pH, the bond is very strong, which is relevant to the bioavailability of this metal (Sanz et al. 2009).

Lead belongs to the elements strongly bound in the soil and accumulated at the humus level. After mixing the sludge with the soil, it is subject to sorption by the oxides and hydroxides of Fe and Mn, and organic matter. In the soil environment, it migrates less actively than zinc or cadmium, but like these elements, it is readily taken up by plants (Kabata Pendias and Pendias 2001; Wuana and Okieimen 2011). In our study, the lead content ranged from 0.92 to 1.57 mg/kg D.M. in wheat leaves and from 0.14 to 0.25 mg/kg D.M. for wheat grains. Fungicidal treatments such as F1 and F2 caused less migration of lead in the soil by the plant and thus lowered the migration of this element to the grain (Fig. 1). Pb content in the grain was the highest on the control plots and F1 + H, respectively, 0.21 and 0.25 mg/kg D.M. The obtained values were above the limits for concentration of the maximum level 0.20 mg/kg D.M. described in the Commission Regulation (EU) No. 1881/2006, and the grain from the plots should not be directed at consumption targets and feed. However, according to Zaccone et al. (2010), lead in cereal grains is localized mainly in the outer layer, and it is eliminated during the process of milling grain to obtain flour (Safari et al. 2015).

According to Zhao et al. (2001), on the soil enriched with sludge, there is an increase of activity of microorganisms in the rhizosphere zone which may lead to an increased solubility of Zn and hence greater absorption of this element by the plants. In our study, plant protection treatments had a significant influence on the zinc content in aboveground parts of spring wheat. The content of Zn ranged from 33 to 45 mg/kg D.M. in wheat leaves and from 0.03 to 0.11 mg/kg D.M. for wheat grains. The largest amount of Zn was taken up by the plants from the plots where one applied fungicidal treatments F1 and F1 + H, which influenced the content of Zn in grain and plants. Their values were higher by approximately 10 and 12%, respectively, in comparison with the control plots (Fig. 1). The zinc belongs to metals, which can be accumulated in the plants, even in large amounts (above 1%) with no apparent signs of toxic effects. It is also worth noting that grass plants which also include grain are the most susceptible to deficiency of this nutrient. Deficiency of this element impairs the synthesis of tryptophan, which in turn can lead to the inhibition of plant growth and influence the final yield (Wuana and Okieimen 2011; Impa et al. 2013).

The statistical analysis revealed correlations between metal concentration in the soil and their concentration in the plants’ aboveground parts. Nickel in the soil was correlated with Cd (*r* = 0.33), Zn (*r* = 0.45), and Cu (*r* = 0.39) in plants, and the Cr content in the soil was significantly correlated with the level Pb (*r* = 0.46) in plants. A positive correlation between the metals showed that all the analyzed metals were taken up by plants from the soil solution in a similar way, which could have a significant influence on plant growth and crop quality.

### Bioconcentration factor for heavy metal in wheat cereals

**Fig. 2:**
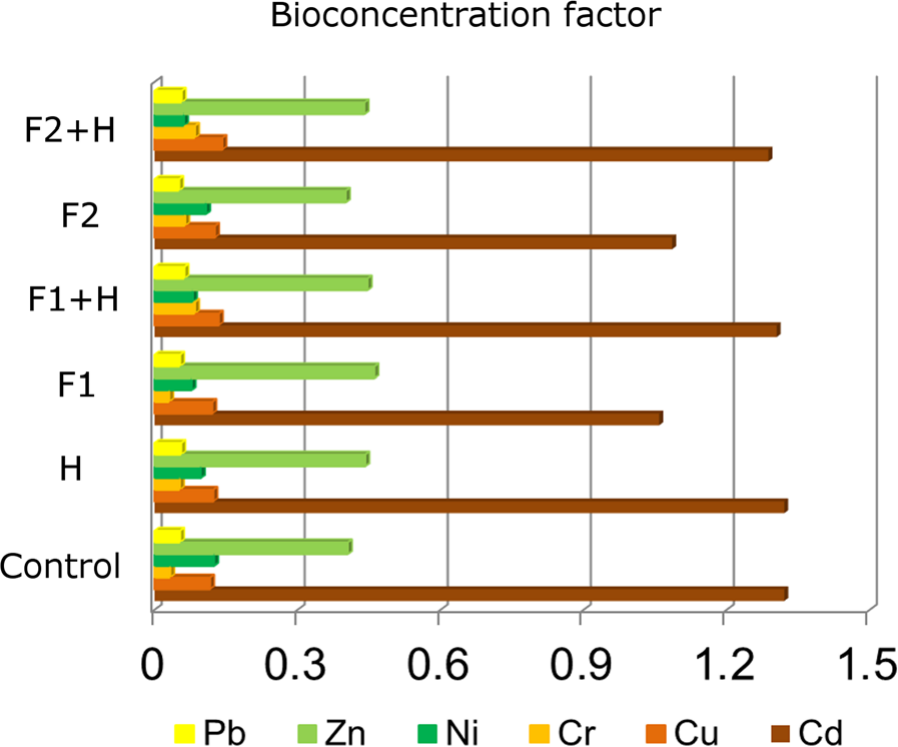
Bioconcentration factor for heavy metals (Ni, Cu, Cr, Cd, Pb, and Zn) in wheat depending on the plant protection treatment

In our study, the calculated value of the BCF allowed for evaluating the ability of wheat to collect heavy metals present in the soil and determine the rate and the migration of metals from the soil solution containing sludge to the aerial parts of wheat depending on the chemical protection variant (Fig. 2). Moreover, the metals’ presence and their toxicity will largely depend on the functions which then perform in the metabolic processes in the organism and susceptibility of the plants to bioaccumulation (Bose and Bhattacharyya 2008).

In the present research experiment, one observed the effect of the applied protective operation on the rate of bioaccumulation. The lowest rate of bioaccumulation for all the analyzed metals was found on the plots with fungicide treatments F1 and F2, while the highest BFC on the control plots and herbicide treatments (Fig. 2). The bioconcentration factor for zinc, copper, and lead fluctuated at a similar level (respectively, BCF = 0.44, BCF = 0.12, and BCF = 0.07) for almost all plots regardless of the variant of used plant protection products. Soil pH also has influence on the bioconcentration of Zn and Cu, since it decreases with increasing pH of soil solubility (Wuana and Okieimen 2011; Wołejko et al. 2013). However, the anionic complex connections of organic mineral exhibit high mobility as an alkaline medium which may also affect the bioconcentration of these metals in plants (Arnot and Gobas 2006). In addition, the highest rate of bioconcentration observed for cadmium in the control plots, with H and F1 + H and F2 + H, was 1.3. Wuana and Okieimen (2011) reported that the rate of bioaccumulation of various heavy metals, especially cadmium, is characterized by high species variety and even varietal diversity. Although cadmium is not necessary for plant development, it is absorbed very easily, both through the roots and leaves, generally in proportion to the concentration in the environment (Gill and Tuteja 2011). It is also important to know bio-chemicals changes in the vicinity of the roots, because they affect the transfer of metal ions to plants and living organisms (Martin et al. 2004).

### The influence of heavy metals on the parameters of wheat quality

The yield of cereals depends largely on the smooth process of photosynthesis and distribution of assimilates during the growing season, which plays a key role in supplying the plant with macro- and micronutrients (Aliyev 2012). Table 5 shows the effect of fungicide-herbicide treatments on the quality parameters of spring wheat grain. The methods used to protect plants caused a slight increase of the protein and gluten content. The sedimentation index was slightly higher as compared with controls. An important indicator of the size and quality of the wheat crop is the number of ears per unit area and the number of grains per spike (Stagnari et al. 2013). In our study, one used treatment H, F1 + H, and F2 + H which contributed to the increase in yield, number of grains per spike, and ears per unit area. In addition, the highest values were observed for thousand grain weight and test weight with the applied treatments F1 and F1 + H (Table 5).

**Table 5:** Effect of different variants of chemical treatments on the quality parameters of spring wheat grain

	NS	NG/S	TGW	GY	TW	GPC	WGC	SC	ZSV	EC
C	213.41	4712.67	36.94	4.60	74.07	9.65	18.13	72.68	23.19	3.03
H	220.25	5037.42	36.56	4.80	73.14	10.08	19.28	72.43	25.23	2.81
F1	199.92	4485.58	39.70	4.63	76.23	9.70	18.25	72.59	23.91	2.45
F1 + H	214.00	4879.08	40.25	5.05	76.23	10.11	19.38	72.44	25.82	2.57
F2	196.58	4367.75	38.62	4.52	73.72	9.81	18.55	72.57	23.96	2.73
F2 + H	210.25	4950.08	38.35	4.92	72.81	9.87	18.64	72.65	24.16	2.66

*C* control, *NS* number of spike, *NG*/*S* number of grains per spike, *GY* yield (t/ha), *TGW* thousand grain weight (g), *SC* starch content (%), *GPC* grain protein content (%), *EC* ergosterol content (mg/kg), *WGC* wet gluten content (%), *ZSV* Zeleny sedimentation value (ml), *TW* test weight (kg/hl)

Table 6 presents the analysis of the Spearman rang correlation of various metal concentrations in grain and grain quality parameters of spring wheat. The statistical analysis showed a significant correlation between the metals contained in grain and the quality parameters of wheat grain. The total content of Cu in grains was positively correlated with the protein and gluten content as well as with the sedimentation value (respectively, *r* = 0.49, *r* = 0.50, *r* = 0.57). Positive correlations were observed between Cd and the yield, the number of grains and ergosterol content (respectively, *r* = 0.41, *r* = 0.55, *r* = 0.56), and Zn with thousand grain weight (*r* = 0.50) at a *p* ≤ 0.05. Positive correlation between these elements in grain and grain quality parameters suggests that each of these metals may influence grain quality parameters. Both Zn and Cu are elements essential for proper plant growth and development. The appropriate contents of these trace elements may positively influence the accumulation in the grain components such as the protein and gluten content as well as the sedimentation value. In turn, Cd is not needed by plants for their development; however, in case of small concentration, no harmful effects on plant growth and development are observed.

**Table 6:** Spearman’s rang correlation analysis concentrations of metal in grain versus grain quality parameters in spring wheat

	Ni	Cd	Cu	Zn	Pb	Cr
NS	−0.29	0.38	0.35	0.09	0.34	0.36
NG/S	−0.13	0.55*	0.26	0.34	−0.02	0.38
TGW	−0.14	0.02	0.13	0.50*	−0.22	0.21
GY	−0.09	0.41*	0.19	0.41	−0.17	0.32
TW	−0.28	−0.06	0.28	0.03	0.38	0.24
GPC	−0.39	0.05	0.49*	0.01	0.34	0.39
WGC	−0.38	0.11	0.50*	0.07	0.22	0.39
SC	0.36	0.20	−0.40	0.24	−0.57*	−0.24
ZSV	−0.51*	0.08	0.57*	−0.09	0.46	0.41
EC	0.06	0.56*	0.10	−0.16	−0.16	0.12

*NS* number of spike, *NG*/*S* number of grains per spike, *GY* yield (t/ha), *TGW* thousand grain weight (g), *SC* starch content (%), *GPC* grain protein content (%), *EC* ergosterol content (mg/kg), *WGC* wet gluten content (%), *ZSV* Zeleny sedimentation value (ml), *TW* test weight (kg/hl)

*Significant correlations for *p* < 0.05

Statistically significant negative correlations were obtained between Ni and the sedimentation value (*r* = −0.51) and between Pb and the starch content in the grain (*r* = −0.57) at a *p* ≤ 0.05. In our studies, the lead concentration in grains was above the allowed norms, so the high amount of this metal may adversely affect the grain quality parameters including starch content.

## Conclusions

1. The concentrations of heavy metals in wheat leaves and grains depended on variants of chemical protection.
2. Protection treatments with fungicide and herbicide conducted during the growing season did not result in exceeding the allowed concentration of cadmium in wheat grain.
3. In the grains from the plots protected with the combination F1 + H, lead content exceeded the maximum levels; it can migrate in the system soil-plant-cereals and be absorbed more easily.
4. The bioconcentration factor for individual metals was dependent on the type of protective treatments and it was the highest for cadmium. This confirmed its high mobility and the possibility of its adverse influence on quality of the final yield.
5. Occurring in soil with sewage sludge, metals significantly affect the qualitative and quantitative parameters of spring wheat.
